# Architecture-Level Optimization on Digital Silicon Photomultipliers for Medical Imaging

**DOI:** 10.3390/s22010122

**Published:** 2021-12-24

**Authors:** Franco Bandi, Victor Ilisie, Ion Vornicu, Ricardo Carmona-Galán, José M. Benlloch, Ángel Rodríguez-Vázquez

**Affiliations:** 1 Organisation Européenne Pour la Recherche Nucléaire, Experimental Physics Department, Esplanade des Particules 1, 1211 Meyrin, Switzerland; 2 Escuela de Ciencias, Ingeniería y Diseño, Universidad Europea de Valencia, Passeig de l’Albereda, 7, 46010 Valencia, Spain; 3 Silicon Austria Labs, Frontend Integrated Circuits & Systems and RF Systems, Europastraße 12, 9524 Villach, Austria; ion.vornicu@silicon-austria.com; 4 Instituto de Microelectrónica de Sevilla (IMSE-CNM), CSIC-Universidad de Sevilla, 41092 Sevilla, Spain; rcarmona@imse-cnm.csic.es (R.C.-G.); arodri-vazquez@us.es (Á.R.-V.); 5 Institute for Instrumentation in Molecular Imaging (I3M), CSIC-Universitat Politècnica de València, 46022 Valencia, Spain; benlloch@i3m.upv.es

**Keywords:** single-photon detectors, digital silicon photomultipliers (dSiPM), single-photon avalanche diode (SPAD), positron emission tomography (PET), Monte Carlo simulations

## Abstract

Silicon photomultipliers (SiPMs) are arrays of single-photon avalanche diodes (SPADs) connected in parallel. Analog silicon photomultipliers are built in custom technologies optimized for detection efficiency. Digital silicon photomultipliers are built in CMOS technology. Although CMOS SPADs are less sensitive, they can incorporate additional functionality at the sensor plane, which is required in some applications for an accurate detection in terms of energy, timestamp, and spatial location. This additional circuitry comprises active quenching and recharge circuits, pulse combining and counting logic, and a time-to-digital converter. This, together with the disconnection of defective SPADs, results in a reduction of the light-sensitive area. In addition, the pile-up of pulses, in space and in time, translates into additional efficiency losses that are inherent to digital SiPMs. The design of digital SiPMs must include some sort of optimization of the pixel architecture in order to maximize sensitivity. In this paper, we identify the most relevant variables that determine the influence of SPAD yield, fill factor loss, and spatial and temporal pile-up in the photon detection efficiency. An optimum of 8% is found for different pixel sizes. The potential benefits of molecular imaging of these optimized and small-sized pixels with independent timestamping capabilities are also analyzed.

## 1. Introduction

**Figure 1:**
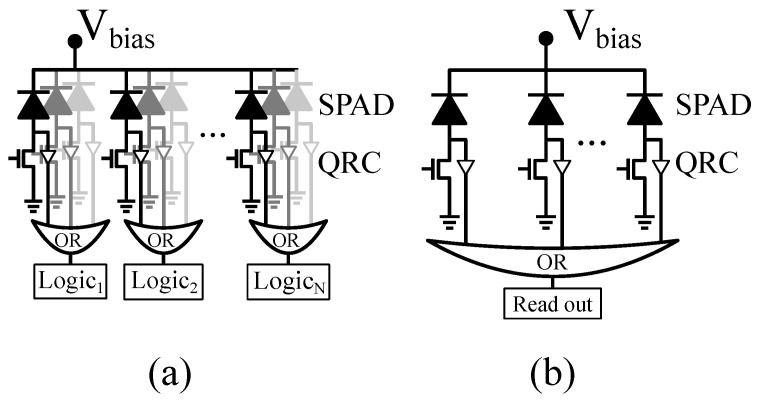
(**a**) Segmented digital silicon photomultiplier (SiPM) and (**b**) digital SiPM with common readout.

Sensors based on single-photon avalanche diodes (SPADs) are nowadays employed in a wide variety of single-photon counting and fast-timing applications, e.g., high-energy physics [1,2]; time of flight (TOF) ranging and 3D imaging [3]; Raman spectroscopy [4]; and bio-medicine, including fluorescence-lifetime imaging microscopy [5,6] and positron emission tomography (PET) [7,8,9], to name a few. When implemented in CMOS image sensor technologies, SPAD sensor architectures benefit from the combination of per-pixel and per-chip processing and control circuitry with good-enough photodetectors [7,10,11]. Particularly, as Figure 1 illustrates, digital SiPMs employ micro-cells consisting of SPADs and embedded processing circuitry to directly encode SPAD avalanches into digital values, thus providing large flexibility for system implementation [12].

**Figure 2:**
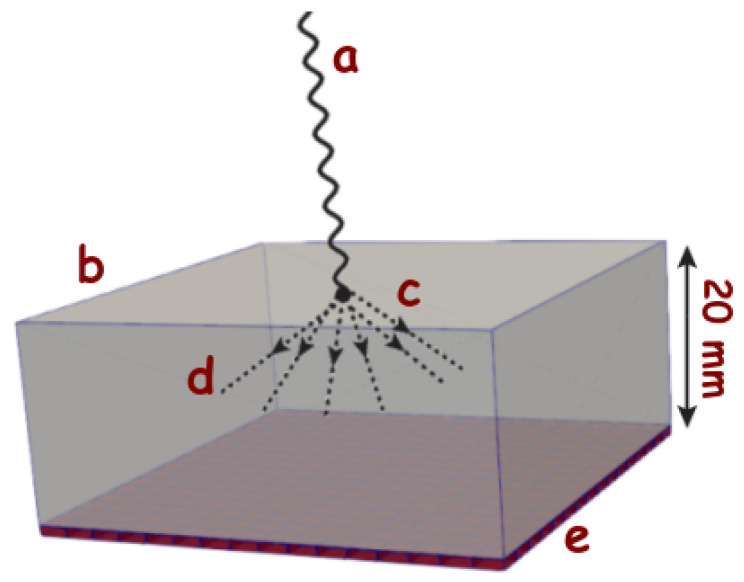
Schematical representation of an incident gamma ray (a) that interacts with a scintillating crystal (b) of typical size (50 × 50 × 20 mm${}^{2}$) at the point (c), producing optical photons (d) (which are of the order of $10^{5}$/MeV depending on the crystal) that are detected by the photodetector placed at (e) that is optically coupled (i.e., with optical grease) to the scintillator.

SiPMs are the most common sensors in PET applications, where they detect the light produced by the interaction between gamma photons and scintillator crystals, as it is schematically shown in Figure 2. Even though for this analysis, we will focus on monolithic crystal blocks attached to an array of SiPMs [13], the issues encountered for this configuration can be partially extrapolated to detectors consisting of pixelated crystals [14,15,16,17]. For a monolithic crystal, the scintillation photons are spread all over the array and, as a result, the amount of photons over each sensor is relatively small. These optical photons contain information about the arrival time of the incident particle, the energy transfer to the crystal, and the first interaction point within the crystal. Therefore, it is essential to maximize their detection to improve the reconstruction of the gamma event and, ultimately, the quality of the PET image.

For pixelated crystal detector configurations, the intrinsic spatial resolution is determined by the pixel’s size. However, as the gamma photon normally suffers from Compton scattering before being totally absorbed, the optical photons are spread over more than one SiPM pixel, similar to the previous case. Hence, for this configuration also, we need a good timing resolution in order to determine the first interaction point and reduce the Compton noise that affects the resolution of the reconstructed medical image. In addition, both pixelated and monolithic detectors require good timing resolution to achieve satisfactory coincidence time resolution (CTR) and enable the inclusion of TOF information for PET scanners. This can be achieved with a smaller SiPM pixel size with independent TDCs.

This paper presents an architectural-level optimization of SiPM for PET under constraints set by pixel non-idealities and fill factor losses, in order to investigate the impact of the nonlinearities introduced by the internal organization of the pixels and the fill factor losses. In the first place, Section 2 briefly presents the trade-offs that have been driving the design of SiPM for PET and SPECT over the last decade. Section 3 provides a general overview of the pixel architecture, including its main parameters and limitations. With an analytical description of the pixel, we propose an architecture-level optimization in Section 4, with an emphasis on the nonlinear response of the sensor and the impact of the SPAD yield. In Section 5, pixels of different sizes are compared using physical simulations. The optimum pixel size for a particular application is obtained. Finally, Section 6 summarizes the most relevant findings obtained during the architecture-level optimization.

## 2. Trade-Offs in SiPM Design for PET and SPECT

**Figure 3:**
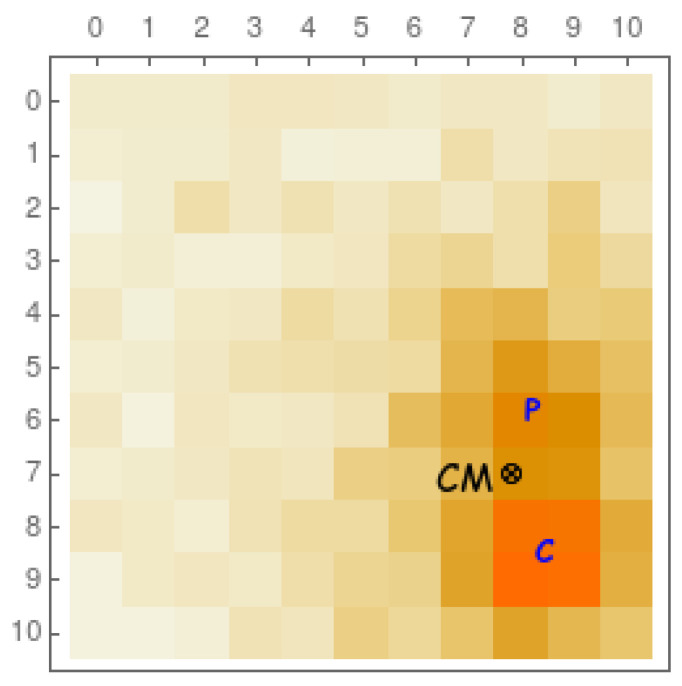
Representation of a 10×10 matrix of 1 × 1 mm${}^{2}$ pixels (coupled to a scintillating crystal as in Figure 2, but with a smaller size) for a typical event with a Compton interaction (C) previous to a photoelectric absorption (P). The electronic readout normally associates the coordinates of this gamma ray event to the center of mass of the energy distribution (CM). Higher-intensity colors correspond to a higher number of detected optical photons.

For PET and SPECT imaging, one needs to reconstruct the interaction point in the scintillating crystal. However, before a gamma ray is totally absorbed through a photoelectric effect, it usually undergoes previous Compton scattering in a non-negligible percentage of cases. In Figure 3, we see (from a Monte Carlo simulation) a typical example of Compton scattering (C) previous to a photoelectric absorption (P). Ideally, in order to perform an accurate image reconstruction, one would *need* to extract the first interaction point (C). However, with current technology, this is a rather challenging task, and the estimated interaction point normally corresponds to the center of mass (CM) of the energy distribution of the optical photon readout. A good timing resolution together with an independent timestamp readout of each pixel would allow us to disentangle the two interactions and their chronological order and therefore recognize the first interaction point. We shall briefly comment on these aspects in the following paragraphs.

The spatial resolution and sensitivity of molecular imaging scanners, such as PET or SPECT, has been drastically improved by reducing the SiPM sizes down to 1 × 1 mm${}^{2}$, reaching depth resolutions down to 0.7–0.8 mm for tomographic images, corresponding to small animal PETs used for pre-clinical applications [18,19]. However, an even bigger challenge is to provide such scanners with a better time resolution and to incorporate TOF information in the image reconstruction algorithm even for standard PET scanners. This can further improve the resolution of the final reconstructed image [20,21], which in turn would allow a patient dose reduction—and all the associated benefits to a lower exposure to gamma ray radiation. Improving the time resolution also helps distinguishing the first interaction point of the gamma photon to push the limits of the depth scanner resolution. This is achieved by reducing the random events leading to false PET coincidences, and the Compton noise represents the gamma ray interactions that take place in the scintillator crystal before it is finally absorbed through a photoelectric effect.

Generally, during the design of SiPMs for single-photon counting applications, such as PET and Čerenkov Telescopes, the photon detection efficiency (PDE) is chosen as a driving figure of merit (FOM). Since PDE is the product of the PDP and the fill factor, designers are forced to implement SPAD pitches from 30 to 60 μm—while keeping the quenching and logic area as small as possible—to achieve fill factors ranging from 50 to 70% [7,22] in order to maximize light detection. The use of PDE as a reference in PET-TOF scanners is further motivated by the fact that the uncertainty in determining the arrival time of the gamma photon is strongly limited by the number of detected photons rather than being dominated by the SPAD jitter or the single shot precision of the time to digital converter (TDC) [23,24]. Despite the relevance of the active area for these sensors, the fill factor losses due to defective SPADs—the ones that must be turned off due to their noise—are not usually considered during the design. This leads to an overestimation of the actual PDE in digital SiPMs.

## 3. Pixel Architecture

As already mentioned, enhancing the PET image quality requires reducing the SiPM size, increasing its PDE and incorporating TOF functionality. We will take this into account as a starting point to define the requirements for our digital SiPM. Then, after establishing the architecture, an analytical expression for the SiPM response will be formulated. With this expression, we will navigate through the design space of the SiPM in Section 4 in order to find the pixel that maximizes the photon detection.

**Figure 4:**
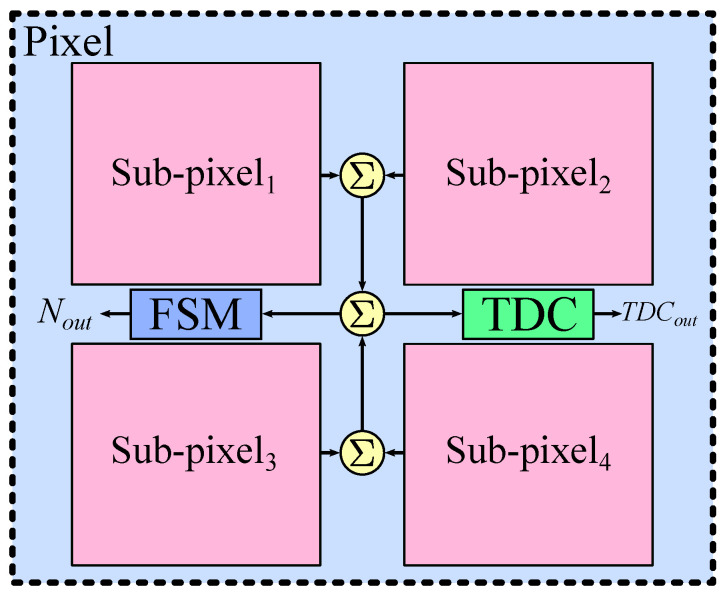
Simplified diagram of a digital SiPM pixel with an adder tree of two levels and the SPADs clustered into four sub-pixels. Each pixel provides both the number of counted photons ($N_{out}$) and the arrival time of the incident particle ($TDC_{out}$).

**Figure 5:**
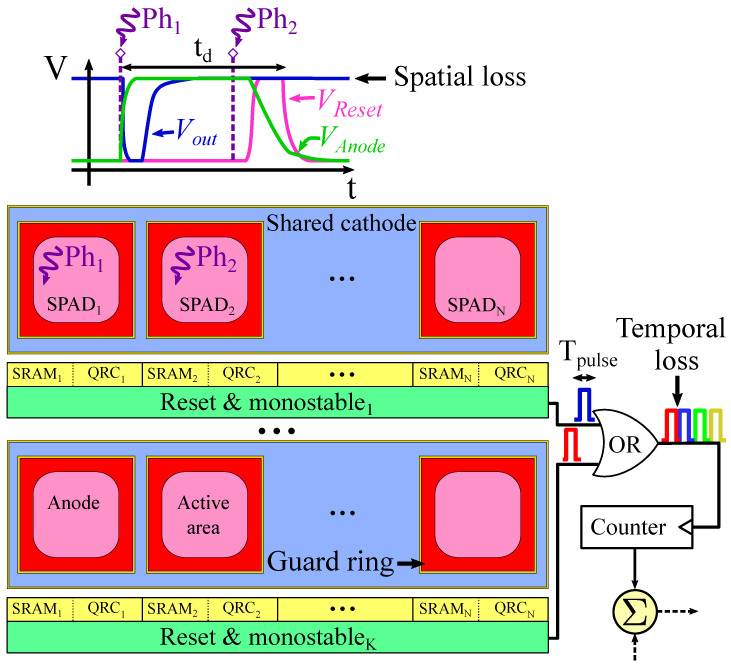
Simplified diagram of a subpixel with *K* microcells connected to a counter through an OR-bus. Each microcell is composed of $N_{\mu c}$ SPADs with their QRCs and SRAMs, a shared reset circuit, and a shared monostable.

For PET applications, we define the term ‘pixel’ as the minimum sensing element that provides information about the scintillation event, i.e., the number of incident photons and their arrival time. The front-end of the pixel is composed by a set of SPADs, which detects the visible photons derived from the scintillation event. These SPADs are driving a counter through an OR-bus, allowing single-photon counting. A finite state machine (FSM) is in charge of processing the scintillation event in order to discard noise. In addition, a TDC is employed to determine the arrival time of the first visible photons as a proxy of the arrival of the gamma photons at the scintillator. Figure 4 illustrates a simplified diagram of the digital SiPM pixel. The FSM and the TDC are clearly identifiable, while the SPADs and the circuits for photon counting are inside each of the four subpixels (see Figure 5).

The analysis of the pixel architecture performed in this paper is mainly focused on the ability of the sensor to count photons. The subpixel block and its operating parameters will determine the major components of the pixel performance. Therefore, the analysis is centered on the subpixel, while the FSM and TDC will be considered as black boxes. As illustrated by Figure 5, a subpixel is composed of several microcells connected to a counter through an OR-bus. Each microcell contains a number of SPADs with their quenching and recharge circuits (QRCs) to quench the avalanche and restore the SPAD to its original photon-sensitive state. In addition, each SPAD is associated with a 1b-SRAM to enable/disable it. Additionally, some common logic is shared among SPADs: a monostable to compress the dead time of the SPADs ($t_{d}$) into a narrower pulse-width ($T_{\mathrm{pulse}}$) to reduce the OR-bus saturation; and a reset circuit to asynchronously reset the microcells once an SPAD has been triggered.

The main idea behind this subpixel architecture is to incorporate some logic circuits that are common either to all SPADs in the microcell or to all microcells in the subpixel. Sharing common logic at different levels will reduce the area not dedicated to photon sensing, thus increasing the fill factor. However, this approach introduces two deliberate sources of inaccuracy, which suppose a penalization on the pixel performance. In the first place, as illustrated by the top part of Figure 5, this topology suffers pile-up if several photons are detected in the same time window ($t_{d}$) by SPADs belonging to the same microcell. The problem is that only one monostable pulse can be generated at a time. The second event of the two nearly coincident detections goes unnoticed. This is usually called spatial compression, since the microcell is behaving as a bigger SPAD composed of $N_{\mu c}$ smaller SPADs [25]. The probability of having pile-up at the microcell level ($P_{\mathrm{sp}}$)—detecting two or more photons in the same microcell—can be estimated using a Poisson distribution [25]:

(1) $$\begin{array}{cc} P_{\mathrm{sp}} & =1-\exp\left(-N_{\mu c}\cdot A_{\mathrm{aspad}}\cdot D_{\det}\right) \end{array}$$

where $A_{\mathrm{aspad}}$ is the SPAD active area, $N_{\mu c}$ is the number of SPADs per microcell, and $D_{\det}$ is the number of detected photons per square millimeter, during the scintillation build-up window that extends from t=0 to $t=T_{\mathrm{clk}}$, through the following equation:

(2) $$\begin{array}{cc} D_{\det} & =\mathrm{PDP}\cdot D_{\mathrm{ph}}\left[1-\exp\left(-\frac{T_{\mathrm{clk}}}{\tau_{\mathrm{sct}}}\right)\right] \end{array}$$

where $D_{\mathrm{ph}}$ is the density of impinging photons, i.e., incident photons ($N_{\mathrm{ph}}$) per pixel area ($A_{\mathrm{pxl}}$) and $\tau_{\mathrm{sct}}$ is the scintillator time constant. It can be estimated performing physical simulations with Geant4 [26] or their toolkits: GATE or GAMOS [27,28]. At first glance, spatial losses could be decreased by reducing $N_{\mu c}$ or $A_{\mathrm{aspad}}$. However, this will have a negative impact in the fill factor, which will be discussed in Section 4.2.

Secondly, at the next level, the probability of pile-up at the OR-bus ($P_{\mathrm{tp}}$)—the overlapping of two or more monostable pulses that become indistinguishable by the counter—can also be estimated with a Poisson distribution [25]:

(3) $$\begin{array}{cc} P_{\mathrm{tp}} & =1-\exp\left(-\frac{N_{\mathrm{spad}}\cdot Y\cdot A_{\mathrm{aspad}}\cdot D_{\det}\cdot T_{\mathrm{pulse}}}{M_{\mathrm{spl}}\cdot T_{\mathrm{clk}}}\right) \end{array}$$

where $N_{\mathrm{spad}}$ is the total number of SPADs in the pixel, $T_{\mathrm{pulse}}$ is the monostable pulse width, $M_{\mathrm{spl}}$ is the number of subpixels per pixel, and *Y* is the SPAD yield, i.e., the fraction of non-defective SPADs in the array. Similarly to spatial losses, temporal losses could be improved by reducing the ratio of SPADs per subpixel. However, since the digital logic is increased, the fill factor will be reduced. Additionally, the temporal compression has an upper bound, as the monostable pulse width is limited by the RC parasitics of the circuit.

These two components model the sensor saturation and limit its performance. Their magnitudes are a function of the pixel area ($A_{\mathrm{pxl}}$), SPAD active area ($A_{\mathrm{aspad}}$), the number of SPADs per microcell ($N_{\mu c}$), and the number of subpixels per pixel ($M_{\mathrm{spl}}$). These parameters define the design space for SiPM architecture exploration and optimization.

## 4. Pixel Optimization

The proposed architectural exploration uses the sensitivity (*S*) as a reference FOM. By doing so, it maximized not only the scintillation photons but also the prompt photons generated at the very beginning of the interaction between the gamma photon and the scintillator crystal [29]. This sensitivity is a function of the active area and the losses associated to spatial and temporal pile-up. These magnitudes are represented by the parameters $A_{\mathrm{aspad}}$, $N_{\mu c}$, and $M_{\mathrm{spl}}$, respectively, which are the design variables. The sensitivity is defined by the ratio of the counted photons ($N_{\mathrm{out}}$) with respect to the incident photons ($N_{\mathrm{ph}}$):

(4) $$\begin{array}{c} S=S\left(A_{\mathrm{aspad}},M_{\mathrm{spl}},N_{\mu c}\right)\equiv \frac{dN_{\mathrm{out}}}{dN_{\mathrm{ph}}} \end{array}$$

In addition, the sensitivity is similar to the PDE, but it needs to include the inherent nonlinearities of the architecture, i.e., pile-up at microcell and OR-bus levels and the defective SPADs, as described in Section 3 [30]:

(5) $$\begin{array}{cc} S(A_{\mathrm{aspad}},M_{\mathrm{spl}},N_{\mu c}) & =\mathrm{PDE}\cdot Y\cdot \left(1-P_{\mathrm{sp}}\right)\cdot \left(1-P_{\mathrm{tp}}\right) \end{array}$$

where $P_{\mathrm{sp}}$ and $P_{\mathrm{tp}}$ are the spatial losses and temporal losses, which were presented in (1) and (3) and PDE is calculated as the product of the PDP and the fill factor (FF):

(6) $$\begin{array}{cc} \mathrm{PDE} & =\mathrm{PDP}\cdot \mathrm{FF}=\mathrm{PDP}\cdot \frac{N_{\mathrm{spad}}\cdot A_{\mathrm{aspad}}}{A_{\mathrm{pxl}}} \end{array}$$

where $A_{\mathrm{pxl}}$ is the total pixel area and $N_{\mathrm{spad}}$ is the number of SPADs per pixel:

(7) $$N_{\mathrm{spad}}=\left\lfloor \frac{A_{\mathrm{pxl}}-\left(A_{\mathrm{tdc}}+A_{\mathrm{fsm}}+A_{\mathrm{cnt}}\cdot M_{\mathrm{spl}}\right)}{A_{\mathrm{spad}}+A_{\mathrm{qrc}}+A_{\mathrm{sram}}+\frac{A_{\mathrm{rm}}}{N_{\mu c}}}\right\rfloor$$

and where $A_{\mathrm{tdc}}$, $A_{\mathrm{fsm}}$, and $A_{\mathrm{cnt}}$ are the areas of the TDC, the FSM, and the counters plus adder tree of the subpixel, respectively. $A_{\mathrm{spad}}$ is the total SPAD area—which takes into account both the active area and dead area: guard ring and cathode connection, and $A_{\mathrm{qrc}}$, $A_{\mathrm{sram}}$, and $A_{\mathrm{rm}}$ are the areas of the quenching and recharge circuit, the 1b-SRAM, and the shared reset and monostable.

### 4.1. Yield Estimation

**Figure 6:**
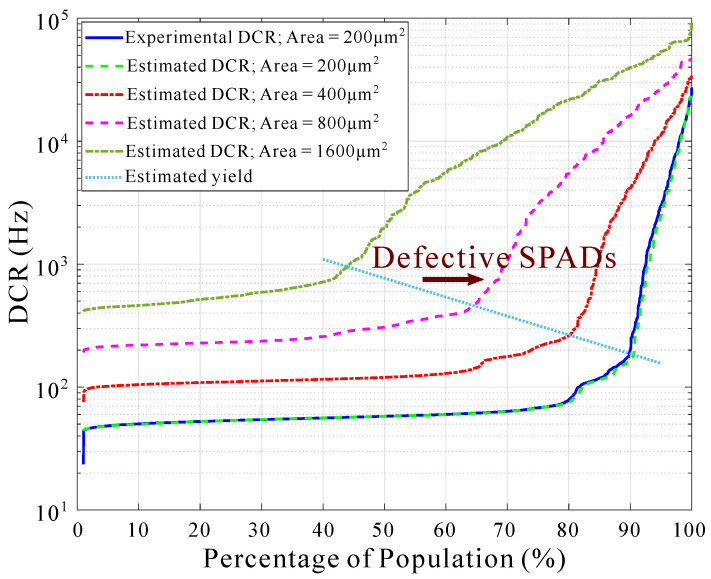
Experimental (solid blue) and estimated (dashed) DCR population distribution for n∼2000 SPADs. Experimental data were obtained from a digital SiPM implemented in LFoundry 110 nm CIS technology. The breakdown voltage is 18 V, the excess voltage is 2 V, and the dead time is 5 μs.

As introduced previously, the actual active area of the SiPM, and hence the sensitivity, depends not only on the SPAD size but also on the SPAD yield. This is because a non-negligible fraction of the implemented SPADs are defective. They suffer from a dark count rate (DCR) that is orders of magnitude higher than the median DCR. These defective SPADs have higher power consumption and can affect neighboring SPADs by crosstalk. Therefore, they need to be disabled. As an example, the solid blue line in Figure 6 illustrates the experimental DCR population distribution obtained from a digital SiPM implemented in LFoundry 110 nm CIS technology, using a P-well/Deep N-well structure [30,31]. This distribution presents a plateau below 100 Hz and a fraction of defective SPADs close to 10%. These noisy SPADs are the result of abrupt variations in the doping concentration and defects in the crystal lattice that lead to higher avalanche probabilities and enhanced photo-emission [32,33,34]. Consequently, it is mandatory to model the SPAD yield as a function of the active area to quantify its effect in sensitivity. This can be done if we assume that these defects are randomly distributed across the different SPADs. The experimental data from [30] can be employed to estimate the DCR of larger SPADs as being composed of smaller SPADs.

Let us consider the vector in Equation (8), which represents the results of the experimental characterization of the DCR for a population of *n* SPADs of size 200 μm${}^{2}$. The estimated DCR of a SPAD with an active area *m* times larger can be obtained as *m* times the root mean square (RMS) value of the experimental DCR for *m* randomly selected *m*-times smaller SPADs, as Equation (9) represents:

(8) $$\begin{array}{cc} {\mathrm{DCR}}_{\exp} & =[{\mathrm{DCR}}_{\exp,1},\cdots ,{\mathrm{DCR}}_{\exp,n}] \end{array}$$

(9) $$\begin{array}{cc} {\mathrm{DCR}}_{\mathrm{est},k}\left(m\cdot A_{\mathrm{aspad}}\right) & =\sqrt{\sum_{i=1}^{m}{\mathrm{DCR}}_{\exp,r_{i,k}}^{2}} \end{array}$$

where ${\mathrm{DCR}}_{\mathrm{est},k}$ is the estimated DCR of the *k*-th SPAD. *k* can take values in the interval [1,n], and the index $r_{i,k}$ represents an element of a m×n matrix composed of uniformly distributed random numbers within the interval [1,n]. All in all, this means that the estimated DCR is composed of *m* randomly chosen experimental values. The solid blue line in Figure 6 represents the experimental DCR for individual SPADs with an active area of 200 μm${}^{2}$, a breakdown voltage of 18 V, an excess voltage of 2 V, and a dead time of 5 μs. The dashed green, red, pink, and dark green lines represent the estimated DCR of SPADs, under the same bias conditions and dead time, with active areas of 200 μm${}^{2}$, 400 μm${}^{2}$, 800 μm${}^{2}$, and 1600 μm${}^{2}$, respectively. The dotted light-blue line represents the yield, which is defined as the border where the DCR rises abruptly, and divides the defective SPADs from the non-defective SPADs. In a practical implementation, these defective SPADs are disabled to reduce the overall SiPM DCR, crosstalk and power consumption.

### 4.2. Optimization Results

The optimization process consists in obtaining the value of $A_{\mathrm{aspad}}$—that stands for the active area of one single SPAD, but it is a proxy of the photosensitive area of the whole SiPM—for which the sensitivity (*S*) is maximized, for each pair of $M_{\mathrm{spl}}$ and $N_{\mu c}$ values. Before starting the optimization process, a set of values needs to be assigned to the variables of Equations (1)–(3) and (6). Table 1 [30] summarizes these values. Then, for detecting the maximum value of sensitivity, we need to solve the following equation:

(10) $$\frac{\partial S(A_{\mathrm{aspad}},M_{\mathrm{spl}},N_{\mu c})}{\partial A_{\mathrm{aspad}}}=0$$

for each pair of possible $M_{\mathrm{spl}}$ and $N_{\mu c}$ values. Notice that $M_{\mathrm{spl}}$ and $N_{\mu c}$ are natural numbers, belonging to $\left\{1,\ldots ,M_{\max}\right\}$ and $\left\{1,\ldots ,N_{\max}\right\}$, respectively, where $M_{\max}$ and $N_{\max}$ are the limits of our exploration of the design space. The results can be grouped in a matrix of this form:

(11) $$\begin{array}{cc} A_{\mathrm{aspad}} & =\left(\begin{array}{cccc} A_{1,1} & A_{1,2} & \cdots & A_{1,N_{\max}}\\ A_{2,1} & A_{2,2} & \cdots & A_{2,N_{\max}}\\ \vdots & \vdots & \ddots & \vdots \\ A_{M_{\max},1} & A_{M_{\max},2} & \cdots & A_{M_{\max},N_{\max}} \end{array}\right) \end{array}$$

Since Equation (10) is a transcendental equation, its closed-form expression is not likely to exist. For this reason, numerical methods have been employed to solve it.

**Figure 7:**
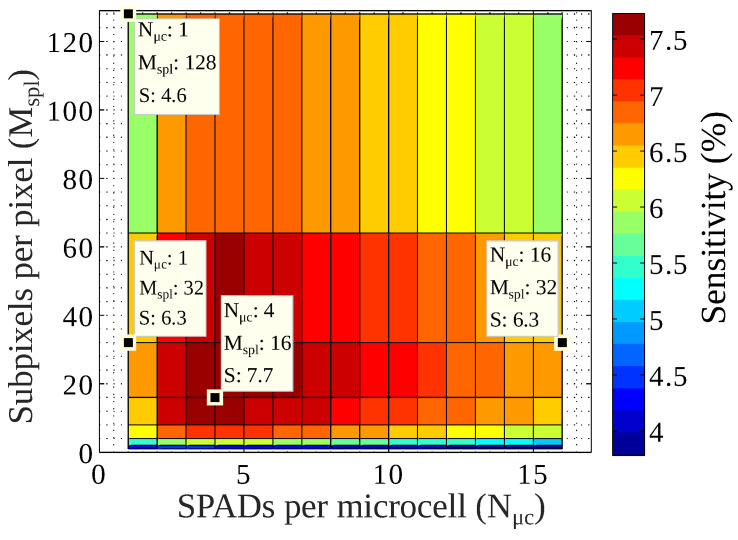
Calculated maximum sensitivity as a function of $M_{\mathrm{spl}}$ and $N_{\mu c}$, for a $1\;mm^{2}$ pixel. The values were obtained from Equation (10), Table 1, and Figure 6. The number of subpixels is a power of 2 in order to maximize the symmetry in the TDC trigger path.

Figure 7 illustrates the calculated maximum sensitivity of a 1 mm${}^{2}$ pixel as a function of the number of subpixels per pixels ($M_{\mathrm{spl}}$) and the number of SPADs per microcell ($N_{\mu c}$). The maximum sensitivity was obtained solving Equation (10), using the data from Table 1 and Figure 6. As expected, sharing some logic between SPADs prevents fill factor losses associated with disabled SPADs; this can be seen if $N_{\mu c}⩾2$. On the other hand, large values of $N_{\mu }c$ impose a large pile-up at the microcells and reduce the sensitivity. In a similar fashion, the figure shows that for the smallest values of $M_{\mathrm{spl}}$, the probability of pile-up at the OR-bus reduces the total sensitivity. Moreover, as soon as $M_{\mathrm{spl}}$ and $N_{\mu c}$ are larger than 2, the sensitivity reaches a plateau with values close to 8%. However, by further increasing $M_{\mathrm{spl}}$, the sensitivity is reduced because the counters and adder tree area are also increased. These results show that the optimization process is able to find the SPAD active area that minimizes the sensitivity losses due to pile-up, defective SPADs, and non-active area, such as guard rings and digital logic.

**Figure 8:**
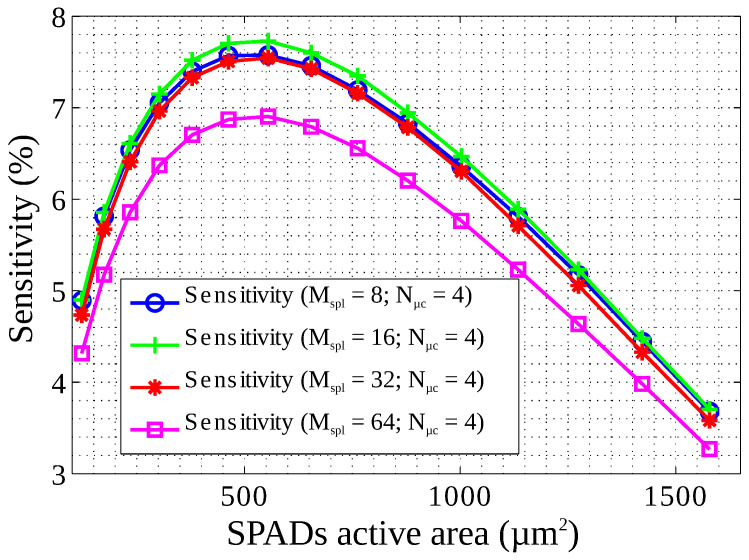
Calculated sensitivity as a function of SPAD active area for a pixel of $1\;mm^{2}$. The values were obtained from Equation (5), Table 1, and Figure 6.

Figure 8 shows the calculated sensitivity as a function of the SPAD active area for a pixel of 1 mm${}^{2}$ and several subpixels per pixel. The sensitivity was obtained solving Equation (5), using the data from Table 1 and Figure 6. This plot shows that the sensitivity is a monotonic function with only one maximum. It is also worth noting that the SPAD active area can be changed slightly without having a large drop in the sensitivity.

Table 2 summarizes the results of pixel optimization for $A_{\mathrm{pxl}}$ = [1, 2.25, 4, 9] mm${}^{2}$. Data were obtained by solving Equation (10), using the data from Table 1 and Figure 6. In all cases, the combination of $N_{\mu c}$ and $M_{\mathrm{spl}}$ yielding the highest sensitivity is reported, together with the sensitivity itself, the SPAD size, the fill factor, and the SPAD yield. In principle, one can think that the sensitivity will increase with the total area of the pixel ($A_{\mathrm{pxl}}$); i.e., the larger the pixel, the more photosensitive area, and the more detected photons. The rational behind that is that for the same architecture, the higher the area, the more photosensitive SPADs, and thus the more active area dedicated to photon detection. However, for a fixed number of SPADs per microcell ($N_{\mu c}$), increasing the number of subpixels ($M_{\mathrm{spl}}$) to cover a larger pixel area introduces higher losses due to temporal pile-up at the OR-bus. Therefore, sensitivity is saturated. In addition, increasing the active area of the individual SPAD ($A_{\mathrm{aspad}}$), in order not to crowd the OR-bus, has a negative effect on the SPAD yield. Therefore, sensitivity saturation is evidenced again. If the technology allowed having a smaller fraction of defective SPADs, the optimization could render larger SPADs, which, in turn, would increase the overall sensitivity. If not, sensitivity is stacked at the same value no matter what pixel size we are considering. It can be observed that the optimization process arrives to sensitivities close to 9%, with independence on the pixel area.

## 5. PET Applications and Simulation Results

In this section, we are going to use Monte Carlo (MC) simulations in order to analyze the timing resolution of a typical PET detector block for different pixel sizes with the corresponding characteristics and performance given in the previous section. This will allow the designers to better understand the space design and the impact of their decisions at the system level.

It has been previously shown that in order to achieve a good timing resolution, one not only needs the lowest value of a timestamp but the intrinsic timing resolution limit can be closely approached by making use of the first N timestamps [35], where N is of the order of 10 (ideally, these first timestamps would correspond to the first scintillated optical photons). In this sense, having timestamp information from each pixel (and not a group of pixels, as currently available on the market) would suppose a significant breakthrough.

In order to optimize the pixel size to detect a high percentage of the first few fast photons (in this case, the first 10), we have performed a GATE/GEANT4 [26,27] MC simulation of a 50×50×20 mm${}^{3}$ LYSO scintillating crystal and incident 511 keV gamma rays with normal incidence. The SiPM of different sizes were placed as previously shown in Figure 2.

In order to model the pixel response to the incident optical photons based on the previous results (resumed in Table 1 and Table 2), we need to make the following considerations. The number of counted photons ($N_{\mathrm{out}}$) comes from the integration of the sensitivity over the number of photons generated in the scintillation process, as can be derived from Equations (4) and (5):

(12) $$\begin{array}{cc} N_{\mathrm{out}} & =\int_{0}^{N_{\mathrm{ph}}}\mathrm{PDE}\cdot Y\cdot \left(1-P_{\mathrm{sp}}\right)\cdot \left(1-P_{\mathrm{tp}}\right)dN^{\prime }. \end{array}$$

By defining α as:

(13) $$\begin{array}{cc} \alpha & =N_{\mu c}+\frac{N_{\mathrm{spad}}\cdot Y\cdot T_{\mathrm{pulse}}}{M_{\mathrm{spl}}\cdot T_{\mathrm{clk}}} \end{array}$$

it follows from Equations (1) and (3) that:

(14) $$\begin{array}{cc} N_{\mathrm{out}} & =\int_{0}^{N_{\mathrm{ph}}}\mathrm{PDE}\cdot Y\cdot \exp\left(-\frac{\alpha \cdot \mathrm{PDE}}{N_{\mathrm{spad}}}N^{\prime }\right)dN^{\prime } \end{array}$$

and from here:

(15) $$\begin{array}{cc} N_{\mathrm{out}} & =\frac{N_{\mathrm{spad}}\cdot Y}{\alpha }\left[1-\exp\left(-\frac{\alpha \cdot \mathrm{PDE}}{N_{\mathrm{spad}}}\cdot N_{\mathrm{ph}}\right)\right]. \end{array}$$

**Figure 9:**
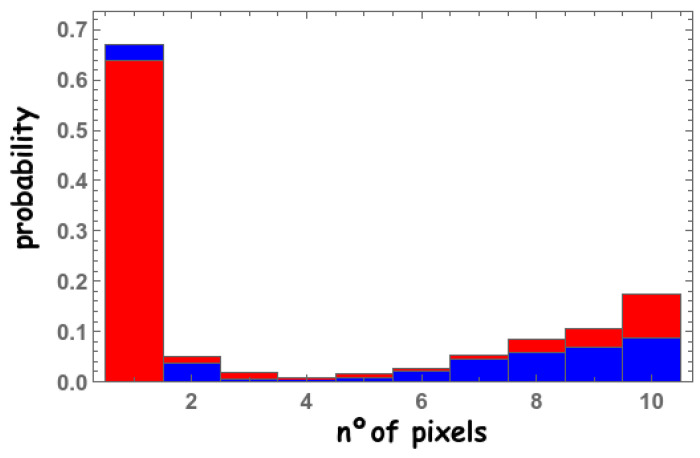
Simulated probability of detecting the first 10 fast photons in up to 10 different pixels for 3 × 3 mm${}^{2}$ (blue) and 1 × 1 mm${}^{2}$ (red) pixels.

The considered pixel for the simulations correspond to those listed in Table 2. Simulation results show that (as one might expect) in order to increase the number of timestamps corresponding to the first 10 detected fast photons, that is, to enhance the probability of detecting these 10 fast photons in different pixels, we necessarily have to use smaller pixels. The probability of detecting the first 10 photons in up to 10 different pixels is shown in Figure 9 for 3 × 3 mm${}^{2}$ pixels (blue) and 1 × 1 mm${}^{2}$ (red). The given results might seem rather counterintuitive. The first reason is that the analyzed events correspond to scintillated photons at different heights within the crystal; as the probability of the gamma ray interaction depends exponentially on the height, the final result is highly nonlinear. A second reason is the low PDE.

**Figure 10:**
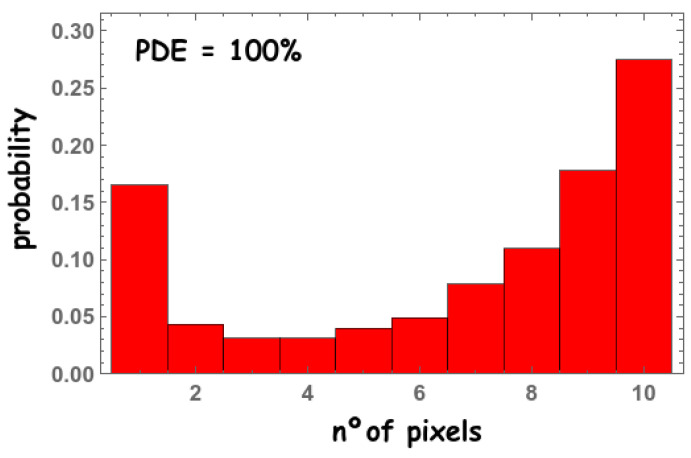
Simulated probability of detecting the first 10 fast photons in up to 10 different pixels for 1 × 1 mm${}^{2}$ pixels for a PDE = 100%.

In order to demonstrate that these two factors introduce non-trivial effects on the fast photon distribution, consider the following ideal case in which the PDE = 100% and the scintillation process takes place close to the upper surface of the crystal. In this case, the fast photon distribution would look like the one shown in Figure 10. Indeed, these results are much more intuitive; as the scintillated photons are originated close to the upper surface, they spread within a greater solid angle, and additionally, all the photons that reach the pixel active area are detected. Hence, the probability of reaching different pixels is much higher.

Thus, we can (roughly) conclude that for similar values of the pixel PDEs, a smaller size pixel would be beneficial in terms of both timing and spatial resolution. Of course, the previous results may vary as they are highly dependent on the experimental setup, i.e., optical treatment of the surface, crystal sizes, etc.; therefore, a similar optimization should be performed for each specific configuration.

## 6. Conclusions

An optimization method for the design of digital SiPMs has been presented. It takes into account the trade-offs between sensitive area, SPAD yield, and nonlinear response. First, a pixel architecture has been defined, and then, its response has been described in analytical form. For the SPAD yield, it has been estimated as a function of the SPAD active area using experimental data.

The presented method helps to identify proper pixel partitioning, that is, the number of subpixels per pixel and the number of SPADs per microcell, in order to maximize the counted photons. Moreover, the optimization process is able to point out the optimum SPAD active area for different pixel sizes. The maximum sensitivity obtained is close to 8% for all sizes. This is a consequence of the similar SPAD sizes that impose almost identical fill factor and yield. It is expected that an improvement in SPAD yield would lead to larger SPAD sizes and better sensitivity.

Finally, we have explored with MC simulations several potential applications with the aim of improving PET scanners performance in terms of both spatial and time resolution and by reducing random events and especially the Compton noise inherent to gamma ray imaging with scintilating crystals.

## Figures and Tables

**Table 1:** Parameter values used for the architectural exploration. The data were obtained from the implemented digital SiPM and its experimental characterization.

Parameter	Value	Units	Description
$A_{\mathrm{pxl}}$	[9, 4, 2.25, 1]	m m ${}^{2}$	Total pixel area
$A_{\mathrm{tdc}}$	6.5 × 10^−3^	m m ${}^{2}$	TDC area
$A_{\mathrm{fsm}}$	14 × 10^−3^	m m ${}^{2}$	FSM area
$A_{\mathrm{cnt}}$	2.6 × 10^−3^	m m ${}^{2}$	Counter and adder tree area
$A_{\mathrm{qrc}}$	72 × 10^−6^	m m ${}^{2}$	QRC area
$A_{\mathrm{sram}}$	18 × 10^−6^	m m ${}^{2}$	SRAM area
$A_{\mathrm{rm}}$	205 × 10^−6^	m m ${}^{2}$	Shared reset and monostable area
PDP	30	%	Photo detection probability
$D_{\mathrm{ph}}$	450	1/mm${}^{2}$	Max. incident photons/sq.mm.
$T_{\mathrm{pulse}}$	800	p s	Monostable pulse width
$T_{\mathrm{clk}}$	20	n s	Clock period
$\tau_{\mathrm{sct}}$	40	n s	Scintillator time constant

**Table 2:** SiPM calculated performance. The data were obtained by solving Equation (10), using the data from Table 1 and Figure 6.

Parameter	9 mm${}^{2}$	4 mm${}^{2}$	2.25 mm${}^{2}$	1 mm${}^{2}$
$[N_{\mu c}$, $M_{\mathrm{spl}}]$	[4,128]	[4,64]	[4,32]	[4,16]
Max. sensitivity (%)	7.8	7.8	7.8	7.7
SPAD size (μm${}^{2}$)	511	512	511	512
Fill factor (%)	41	40	40	39
Yield (%)	77	77	77	77
SPADs per pixel	7217	3101	1747	762

## Data Availability

The data presented in this study are available on request from the corresponding author. The data not contained in the article are not publicly available due to ongoing result protection and technology transference processes.

## Abbreviations

The following abbreviations are used in this manuscript:

PMT	Photomultiplier tubes
SPAD	Single-photon avalanche diode
TOF	Time of flight
CTR	Coincidence time resolution
PET	Positron emission tomography
CIS	CMOS image sensor
PDP	Photon detection probability
SiPM	Silicon photomultipliers
PDE	Photon detection efficiency
FOM	Figure of merit
TDC	Time to digital converter
FSM	Finite state machine
QRC	Quenching and recharge circuits
$t_{d}$	Dead time of the QRC
$T_{\mathrm{pulse}}$	Monostable pulse width
$T_{\mathrm{clk}}$	Clock period
$\tau_{\mathrm{sct}}$	Scintillator time constant
$N_{\mathrm{spad}}$	No. of SPADs per pixel
$N_{\mu c}$	No. of SPADs per microcell
$M_{\mathrm{spl}}$	No. of subpixels per pixel
$A_{\mathrm{aspad}}$	Active area of a SPAD
$A_{\mathrm{spad}}$	Total area of a SPAD
$N_{\mathrm{ph}}$	No. of incident photons
$N_{\mathrm{out}}$	No. of counted photons
*S*	Sensitivity
*Y*	Yield, fraction of non-defectives SPADs
$P_{\mathrm{sp}}$	Probability of pile-up at microcell level (spatial losses)
$P_{\mathrm{tp}}$	Probability of pile-up at OR-bus level (temporal losses)
$A_{\mathrm{pxl}}$	Pixel area
$A_{\mathrm{tdc}}$	TDC area
$A_{\mathrm{fsm}}$	FSM area
$A_{\mathrm{cnt}}$	Counter and added tree area
$A_{\mathrm{aqrc}}$	QRC area
$A_{\mathrm{sram}}$	SRAM area
$A_{\mathrm{rm}}$	Shared reset and monostable area
$D_{\det}$	Density of detected photons
$D_{\mathrm{ph}}$	Density of impinging photons
DCR	Dark count rate
MC	Monte Carlo
